# Semiochemical 2-Methyl-2-butenal Reduced Signs of Stress in Cats during Transport

**DOI:** 10.3390/ani14020341

**Published:** 2024-01-22

**Authors:** Courtney Archer, John McGlone

**Affiliations:** Laboratory of Animal Behavior, Physiology and Welfare, Animal and Food Sciences, Texas Tech University, Lubbock, TX 79409, USA; arche214@umn.edu

**Keywords:** domestic cat, pheromone, interomone, stress, behavior

## Abstract

**Simple Summary:**

Sixteen cats were used in a model of behavioral and physiological transport stress. Cats were not accustomed to being transported. In an objective evaluation, cats wore a PetPace (PP) collar that recorded carotid pulse rate (PR) and general activity. Video cameras recorded cat behavior during the 70 min transport experience. Cats also wore a plastic collar containing either 2-methyl-2-butenal (2M2B) collars or a placebo collar. This randomized, placebo-controlled, blinded study found that cats with a 2M2B collar had a lower PR, slept more, sat less, and self-groomed more compared with cats wearing a placebo collar. Control cats hid near the back of the transport kennel, and some vomited or had excessive salivation, whereas cats with 2M2B collars did not hide, vomit, or salivate. This controlled study demonstrates behavioral and physiological benefits to transported cats from the use of 2M2B collars.

**Abstract:**

Some cats experience stress when they have novel experiences, such as infrequent transport. This study was a randomized, placebo-controlled, blinded study that sought to objectively evaluate the effects of a 2M2B collar on transported cat physiology and behavior. The statistical model included effects of cat treatment (2M2B vs. control), period (70 min), sex, and interactions. Cats wearing 2M2B collars had an 8% lower PR (*p* < 0.01), and they slept more and did not hide at the back of the kennel. While control cats vomited or showed excess salivation, cats with 2M2B collars did not show these signs of stress. Male cats were less active during transport than females. Male cats slept more with 2M2B collars compared with male cats with a control collar, but females showed similar sleeping overall regardless of which collar they wore. Female cats increased activity during transport when they had a 2M2B collar, while male activity did not differ with control or 2M2B collars. These data support the concept that the semiochemical 2M2B can reduce stress in transported cats based on objective physiological and behavioral measures.

## 1. Introduction

The domestic house cat (*Felis catus*) is a domesticated, obligatory carnivore that lives in or near homes as a pet or companion around the world and is not much different genetically from its ancient ancestor, the Wildcat [1]. While it is unusual for a predator to be kept as a pet, the domestic cat benefits from its dual role as companion and vermin controller. In some ways, the house cat is living in an environment that contrasts with its natural habitat, which would include hunting areas and environmental surveillance. One must appreciate the olfactory acuity of the domestic cat and how its olfactory environment can be less rich in a home than in the wild [2].

The cat has a well-developed olfactory system that includes the main olfactory epithelium (MOE) and the vomeronasal organ (VNO) [3]. Both systems are functional in the cat. The MOE perceives primarily aerosol and volatile molecules, while the VNO can be activated by liquid or less-volatile chemical signals. A semiochemical is a broad term used for olfactory signals that can change behavior and can include pheromones, interomones, attractants, and plant products that have an olfactory-behavior effect. Some semiochemicals activate one or the other olfactory systems (MOE or VNO). Catnip, for example, activates the MOE and not the VNO to induce its behavioral effects [4]. The use of semiochemicals to reduce stress is a recent idea that should be possible because the olfactory sensory system has neural links to areas of stress control in the amygdala and hypothalamus [3]. 

Most semiochemicals and pheromones can be found naturally in the environment. 2-methyl-2-butenal (2M2B) is a natural molecule found in many plants, animals, and foods. The 2M2B molecule is a food-grade flavoring agent and is safe if consumed by humans [5]. 2M2B is found in berries, certain vegetables, chicken fat, butter fat, beer, and the mammary secretions of rabbits [6]. First identified as a rabbit pheromone, 2M2B is a volatile molecule that rabbit pups use to orient towards their mother’s nipple, inducing nipple-searching behaviors [6,7]. While not described as a pheromone other than in the rabbit, 2M2B has effects on the brain and behavior of many species. Since semiochemicals are conserved across species, one compound may invoke different behavioral or biological changes in different species. Studies have reported that 2M2B can change the brain and/or behavior of dogs [8], cats [9], pigs [10], and humans [8]. Clearly, the metabolic production of 2M2B and its perception by olfaction are conserved over many species. Generally, 2M2B at low concentrations has a calming effect on animals (as an interomone) in that when animals can smell it, especially during stressful periods, they have behavioral and physiological signs of reduced stress [11]. While the interomone 2M2B has not been shown to be a pheromone in cats, this molecule can reduce aggression and measures of anxiety in paired cats [9].

The interomone effect refers to when a semiochemical has a pheromone effect on the physiology or behavior of one species and is not described as a pheromone in a second species, but the molecule(s) have effects on the behavior or physiology of the second species [12]. For example, 2M2B has not been reported in the secretions of pigs but serves as an interomone in that it increases feed intake and weight gain in healthy, weaned pigs [10]. Another interomone example is the pig pheromone androstenone, which has been reported to stop barking in dogs [13].

Transportation of companion animals is unavoidable in many situations (e.g., veterinarian clinic visits). Transportation becomes a stressful event for both the owner and the animal. Cats are less often transported than dogs, so the novelty of transport may be stressful to the domestic house cat, as has been shown for other species [14]. Behaviors such as vocalizing, increased defecation and urination, and ears pressed back are indicators of stress that cats express during all stages of a veterinary visit, especially the transport phase [15]. The EU has regulations about the transport of cats, but a review of the literature found insufficient data to support evidence-based regulations [16], other than to recommend conditioning cats to transport crates and procedures. 

The use of semiochemicals in cats to minimize common stressors has not been widely studied. Models of transport stress, while common in livestock, have not been often described for domestic cats. The use of different feline semiochemicals, such as feline facial semiochemicals, has been reported to have calming effects [17] and may reduce urine marking [18]. A feline interdigital pheromone has also been reported to aid in correcting inappropriate behaviors, such as scratching [19]. No study to date has provided clear evidence that the molecules marketed as cat pheromones are actually pheromones in cats.

Feline facial “pheromones” have been reported for cats [2]. While marketing calls these molecules “pheromones”, it is important to point out that studies have not confirmed that they are pheromones according to thoughtful reviews and papers that set criteria for calling a molecule(s) a pheromone [7,11,20]. Here we refer to the cat facial molecules as semiochemicals because they can change behavior but have not been shown to be pheromones by accepted scientific definitions backed by appropriate studies. 

One recent study developed a transport model for cats in order to evaluate the effects of a synthetic semiochemical as a potential intervention for feline stress during transportation. Shu and Gu [21] conducted a pilot study to examine if the Feline Facial Semiochemical (FFS) might provide a stress-reduction effect during short-distance transport of cats. Data were collected by cat owners, not validated observers. Cat owners were asked to spray either a placebo or a cat facial semiochemical in a cat carrier and then transport their cat(s). They evaluated 150 cats (75 per treatment group). At baseline time zero, baseline stress scores did not differ between the two groups. FFS-treated cats were less active during transport than control cats. Both control and FFS-treated cats meowed less over time, but the reduction was greater among FFS-treated cats. The authors used a visual analog scale (VAS) as a key measure of stress response. The effects of treatment were not significantly different (*p* = 0.755) on the VAS overall between the treatments. When baseline data were used as a covariate, the treatment effect was significant among cats with higher stress scores. Although the effects of FFS were small, one might expect it to work better for cats that were more stressed. Here, we used a similar transport model to assess the effects of an interomone on cat behavior and physiology. Most previous work did not include objective measures of physiology (such as heart rate). This study was designed to sample both the behavior and physiology of transported cats using objective measures in a controlled setting (not in homes).

This work had one primary objective: To determine if 2M2B can be used to change behavior and physiology to improve the welfare of cats during transport. We used measures of cat behavior and heart rate to evaluate cat experiences. 

## 2. Materials and Methods

All research was conducted at Texas Tech University (TTU) with approval by the TTU Animal Care and Use Committee (IACUC # 19104-12). All procedures were consistent with the U.S. Animal Welfare Act.

### 2.1. Animals 

Sixteen mixed-breed cats (8 males and 8 females) both intact and neutered within the age range of 1.5 to 16 years of age (5.0 ± 4.3) were used in this study (cat characteristics are given in Table S1 in the Supplementary Material). Cats weighed between 2.6 and 4.7 kg (3.91 ± 0.71). All cats were selected from a USDA-inspected and certified facility. Cats were fed once daily with ad libitum water, except during transport. Cats selected for the study had not experienced transportation or carriers within 5 months of the start of the study. Cats were individually penned in the research facility with concrete floors, a resting board, and a chain link fence between adjacent cats. Each pen measured about 1 × 4 m. Cats received environmental enrichment and daily human contact, with minimal human handling. Cats infrequently left their home pen, and cats were not acclimated to transport. 

### 2.2. Experimental Design 

**Figure 1:**
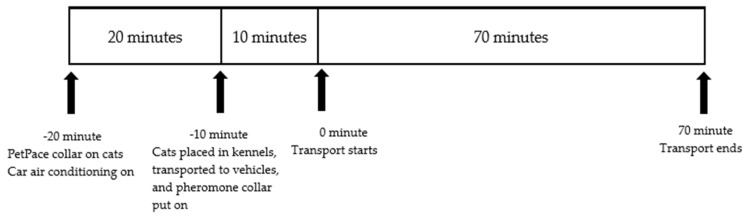
Timeline of experimental procedures.

Sixteen cats were transported individually in four separate trips over 70 km within 70 min (Figure 1). For all trips, cats were transported in pet kennels with dimensions of 0.71 m × 0.52 m × 0.55 m. One trip consisted of four vehicles, each transporting one cat within a pet kennel. A trip was considered the distance to and from a park in Vernon, Texas, that was estimated to be 70 km away. The four vehicles were split into two groups to represent the two treatments. Two cars were the control (no pheromone), and the other two cars were the 2M2B (pheromone). The cars remained in their designated treatment groups for all four trips to avoid the exposure of control cats to the 2M2B pheromone. 

The treatment or placebo collars were placed on the cats for a 10 min acclimation period before transportation began. Cats wore the PetPace monitor and collar throughout the transport period. During transport, drivers maintained a relatively consistent speed (~112 km/h) with minimal sudden turns or stops. The U-turn of the round trip occurred 30–40 min into the travel time. Temperature was maintained at around 25 °C for the duration of the trip. No food was in the vehicles during transportation. Noise was mitigated as much as possible; no music was playing, and no researchers spoke during the transport. All noise capable of being controlled by the researchers was diminished in the vehicle.

Each treatment group was balanced for sex, and trips were balanced for treatment. Each of the 4 trips had four vehicles, with two vehicles of control and two vehicles of 2M2B. Each treatment within the trip would have one female and one male cat (i.e., for each trip, *n* = 4 cats, 1 male and 1 female/treatment group). 

Treatments were delivered through collars produced by PeIQ (Omaha, NE & Boise, ID) and contained either the 2M2B treatment or the placebo control treatment that contained nothing. Placebo collars appeared identical to the collars containing 2M2B. We have previously reported the release of 2M2B from these collars [22] over a 4-week period. New collars were used during this work. All cats wore identically shaped collars to blind researchers to each treatment group throughout the study, but they were able to see the two colors of collars (so as not to confuse the 2 treatments). Each treatment was designated a collar color, but researchers were blind to which color represented which treatment. This allowed researchers to verify that trips were balanced by sex and treatment. Thus, this work was a placebo-controlled, randomized study with investigators blind to treatment groups. Trained, validated observers recorded objective behavior data while also being blind to treatment groups.

### 2.3. Measurements 

Measures of behavior and physiology were collected. Heart rate data are rarely reported for cats due to the difficulty of obtaining these measures. Physiological measures (pulse rate and activity) were monitored by a PetPace pulse rate monitor collar from Pet Pace LLC, Burlington, MA, USA. The collar senses carotid artery pulses, which correlate with heart rate; by definition, heart rate equals pulse rate. Cats tolerate a collar well, while more invasive heart rate sensors will limit cat mobility. The PetPace collars record both pulse rate and general activity. To obtain the PetPace data, the collar recordings must be uploaded to the internet and then downloaded for data analysis.

Behaviors were recorded by HDBV-301 video cameras (HausBell, USCLOUND TRADE LTD., Rosemead, CA, USA) placed inside the vehicles. The timeline of the study is shown in Figure 1. The PetPace monitors were acclimated to cats for 20 min before they were placed in the kennel and transported to the vehicles. 

Parameters were recorded every 2 min throughout the trip. The starting and ending times were recorded to match the times of the PetPace monitors. The pulse rate was measured as beats per minute (bpm) as the device rested on the carotid artery. 

Activity level was recorded by the PetPace collars as numbers (ranging from 0.4 to 27.7). Activity data were obtained by averaging values in 10 min intervals during the 70 min trip. Activity data were also collected from live video recordings.

Behavior measures (each defined in Table 1) were collected by trained, blinded, and validated observers from training videos collected during the study. Videos were watched using 1 min scan sampling, and observations of 10 min intervals were averaged to determine the percentage of time animals spent expressing each behavior. The location of the cat was also recorded. Location was determined by the position within the kennel the cat chose to rest or sit in. Location was when the cat head was either at the front or back of the kennel. The location was recorded every minute to determine the percentage of time the cat spent in the two locations. Detailed time of each behavior was used to calculate the time the cat spent adjusting positions and being active based on video recordings. Behaviors are outlined in the ethogram portion of Table 1. 

### 2.4. Statistical Analyses

Data were first evaluated for assumptions in the analysis of variance (ANOVA). For data that met the assumptions for parametric analyses, the data were analyzed with a simple repeated measures ANOVA with two treatments (control vs. 2M2B). Each cat served as an experimental unit. Physiological and behavioral measurements were analyzed using repeated measures in Proc Mixed Procedures of SAS 9.4. The statistical model included fixed effects as treatment, cat within treatment, sex, period, interaction of treatment and period, and interaction of treatment and sex. Period refers to the 10 min intervals within the 70 min travel time. The predicted difference test within the SAS Proc Mixed Procedures was used to separate least squares means when the parameter overall F-value was significant (*p* < 0.05). All cats were adults; cat body weight and age did not interact with treatment effects.

When measures varied over time, multiple regression models were generated to describe and compare the response of PR over time during transport. If the response over time fit a linear, quadratic, or cubic model, a graph was generated to show both the data points and the regression line values. Generally, PR and HR increase during the trip, then they are reduced, perhaps as cats acclimate to the conditions. Best-fit regression models were compared for cats in the control or 2M2B treatment groups. For other measures where the time-by-treatment effect was significant (*p* < 0.05), but with a non-significant regression model to describe the data, a simple line graph was produced showing at which time periods the control differed from the 2M2B treatment group.

For non-parametric analyses, a chi-square test was used, for example, to determine if the location of the cat during transport was different between control and 2M2B-treated cats. Correlation coefficients were calculated to determine the relationship between activity recorded by the PetPace collar and video and pulse rate recorded with the PetPace collar and video to assist future work in the validation of this methodology. 

## 3. Results

Overall, during transport, cats wearing the 2M2B collar had decreased PR (*p* < 0.0001), sitting behavior (*p* = 0.04), but increased sleeping (*p* = 0.006) and self-grooming (*p* = 0.09) compared with cats wearing a placebo collar (Table 2). Cats that wore the 2M2B collars also tended to have a higher activity level as monitored by the PetPace collar (*p* = 0.07); the main difference in activity observed was more self-grooming among 2M2B-treated cats compared with placebo-controlled cats. Other behaviors, such as lying and adjusting positions, were not altered by the 2M2B treatment (*p* > 0.10). Self-grooming was not observed in cats wearing a control collar, while self-grooming was present among cats wearing a 2M2B collar. 

Cats in the 2M2B-treated groups spent more time at the front of the kennel, while control cats spent more time at the back of the kennel (*p* < 0.01; Table 3). This significant effect would be clear to any casual observer. Hiding near the back of the kennel would indicate more fear or discomfort among control cats than those exposed to 2M2B therapy. 

**Figure 2:**
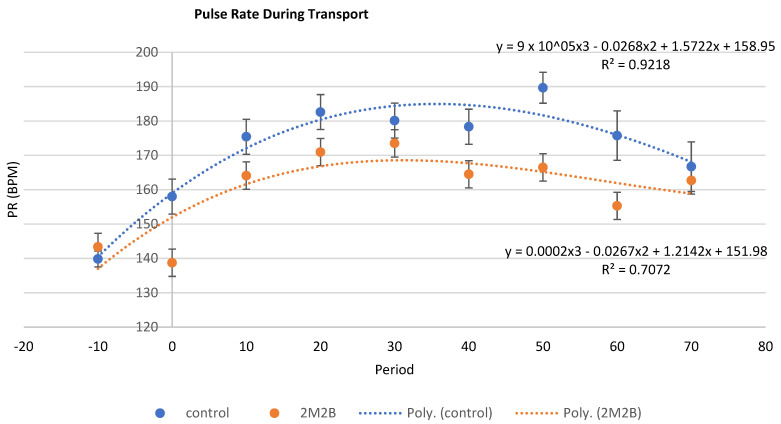
Treatment by time effects (control and 2-methyl 2-butenal (2M2B)-treated (*n* = 8 cats/treatment)) on pulse rate (LS means ± SE, BPM). Cats with control or 2M2B collars had a similar overall response, with elevated then declining PR during transport. Note that although cats in the 2 treatment groups had similar PR at time −10 (prior to collar placement); averaged other times, cats with 2M2B collars had 8% lower (*p* < 0.001) PR than cats with a control collar. The quadratic equations that best describe the response over time for each treatment were consistent with high R^2^ values. *n* = 16 cats; treatment effect, *p* < 0.0001; see Table 2 for statistical details.

Presented in Figure 2 are PR data from control and 2M2B-treated cats over the course of the study. Note that for all cats, their PR increased and then decreased as they continued their journey. The overall lower PR for 2M2B-treated cats compared with control cats was observed to be consistent over each time point (Figure 2). The regression equations for control and 2M2B-treated cats were different, primarily in the lower PR levels of cats wearing 2M2B collars than control cats. Cats in the 2 treatment groups started out with very similar PRs, but the cats with control collars had a uniformly higher PR than cats with 2M2B collars. The drop in PR associated with time zero was when the 2M2B collar was placed and before transport began. While one can see the immediate drop in PR once the 2M2B collar was placed, this did not happen among cats with a control collar (Figure 2). Cats with 2M2B collars had an average of 8% lower PR than control cats overall. 

**Figure 3:**
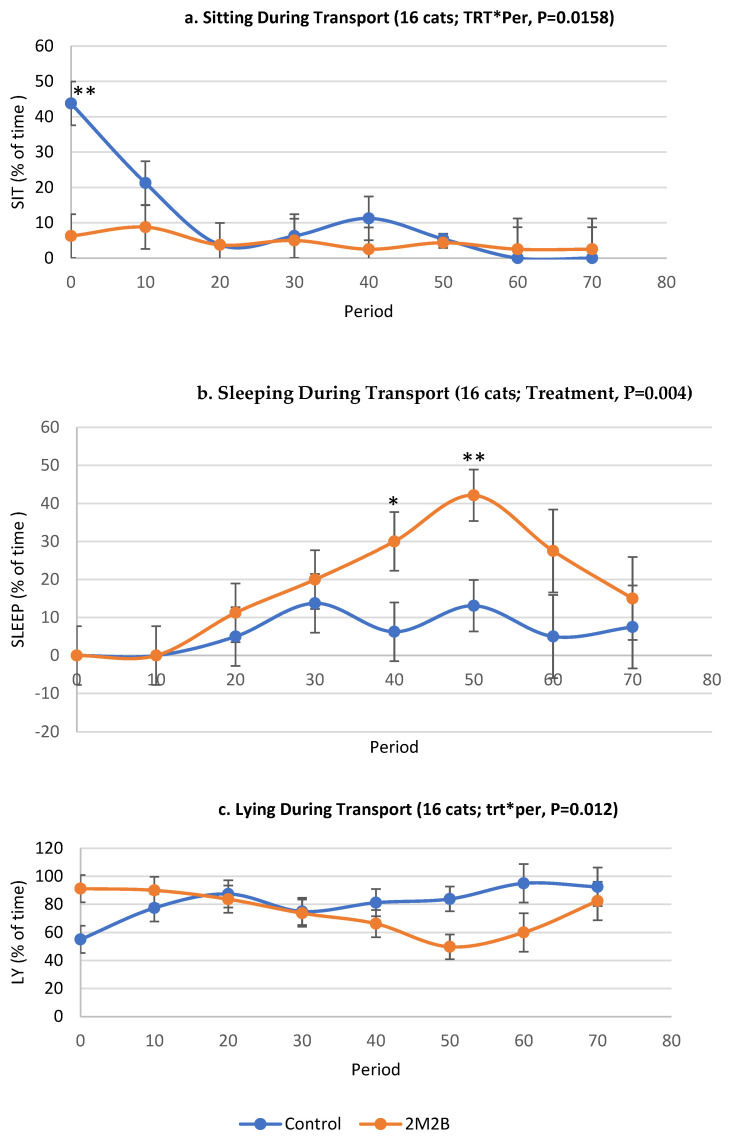
Sitting, sleeping, and lying of cats during transport with control or 2M2B collar. Data were collected via video recordings starting at time zero through 70 min of transport. Treatment by time effects (control (*n* = 8) and 2-methyl 2-butenal (2M2B)-treated (N = 8)) on sleeping (LS means ± SE, % of time). *, **, means difference between the control and 2M2B-treated groups being significant at *p* < 0.05 and *p* < 0.01, respectively. The treatment effect was significant (*p* < 0.01) (2M2B-treated cats slept more than control cats), but the treatment-by-time effect was not statistically significant. See Figure 6 for sex by treatment effect (*p* < 0.001) on sleeping. While the treatment vs. control effect was significant overall, the time points of 40 and 50 min after transport found more sleeping among cats with 2M2B collars than control collars (*p* < 0.05).

Period effects were observed: All cats had increased, then decreased pulse rates (*p* < 0.0001; Figure 3), activity levels (*p* = 0.07; Figure 3), and spent more time apparently sleeping (*p* = 0.004; Figure 3, but had decreased sitting behaviors (*p* = 0.02; Figure 3) over time. The interaction between treatment (2M2B vs. placebo) and period was significant for lying and sitting (Table 3; Figure 3). The treatment by period effect was not significant for sleeping, but the main effect of treatment (*p* < 0.01) showed that cats with 2M2B collars spend more time apparently sleeping during transport than cats wearing a placebo collar.

Cats treated with 2M2B spent more time lying at period “0” (start of the transportation) and “10” (10 min into the travel), but less time lying at period “40” to “70” (40–70 min into the travel time) compared to the control group (Figure 3). These changes document how transported cats adapted to travel in general. 

**Figure 4:**
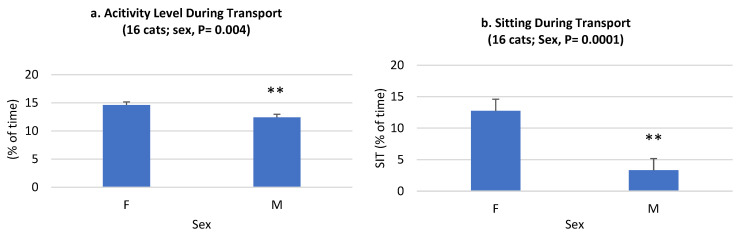
Effects of cat sex during transport (control (*n* = 8) and 2-methyl 2-butenal (2M2B)-treated (*n* = 8)) for activity (**left**) and sitting (**right** graph) behaviors. Activity levels were recorded by the PetPace collars (LS means ± SE, % of time). Sitting behaviors were objectively quantified from video records. **, means difference between the control and 2M2B-treated groups is significant at *p* < 0.01. Note males were less active than females, and they spent less time sitting because they were less active (sitting was considered active, or not resting/sleeping). Male cats were less active, and they sat less than females overall.

**Figure 5:**
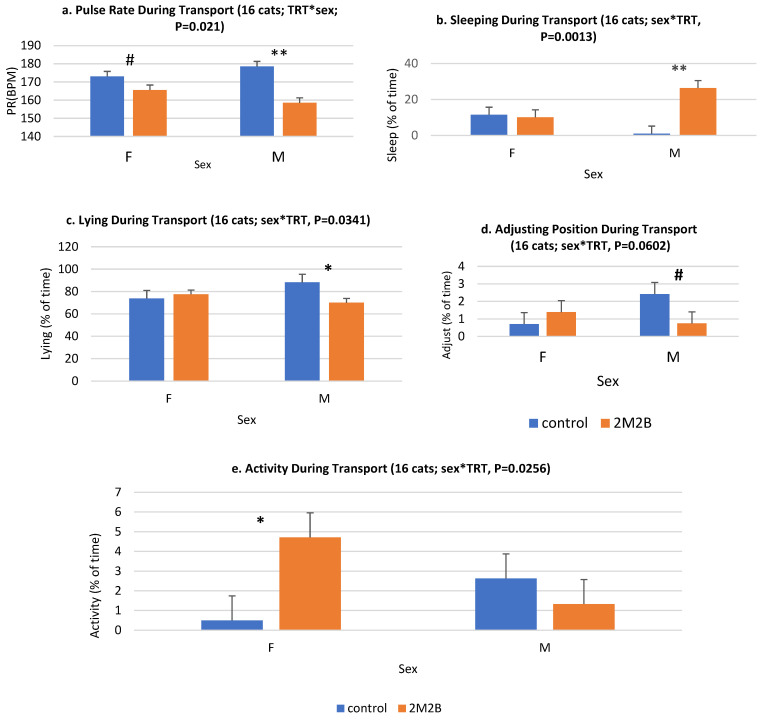
Sex-by-treatment interaction for transported cats’ physiology and behavior. (LS means ± SE, BPM). **#**, ** means difference between the control and 2M2B-treated groups being significant at *p* < 0.10 and *p* < 0.01, respectively. * shows a trend with a *p* < 0.04. Overall, the sex-by-treatment effect was significant (*p* < 0.05) for most measures and showed a trend for adjusting the body during transport (*p* = 0.06). However, PR was lower among both males and females exposed to 2M2B collars compared with cats exposed to control collars. Male cats exposed to 2M2B had a greater PR-reduction effect than female cats. Male cats with 2M2B collars slept more but had less time spent lying and tended to adjust position less during transport. Transported female cats were more active with 2M2B collars than control cats, while male cat activity did not differ between 2M2B and control collared cats.

Some sex and treatment-by-sex effects were observed. Male cats were less (*p* < 0.01) active than female cats, and they spent less (*p* < 0.001) time sitting than females (Figure 4). The sex-by-treatment effects describe how male and female cats responded differently to the 2M2B collars. Both males and females had lower PR when they wore a 2M2B collar than when cats wore a placebo collar; however, the decrease in PR was more pronounced among male cats than female cats (Figure 4). Male cats with 2M2B collars spent more time sleeping, less time lying, and less time adjusting body position. Overall, 2M2B collars increased the activity of females but had no effect on the overall activity of males (Figure 5). We had an insufficient sample size to evaluate the effects of intact vs. castrated animals. Because we did observe sex effects, an examination of the effects of spay/neuter on cat behavior in future studies might generate interesting effects.

**Figure 6:**
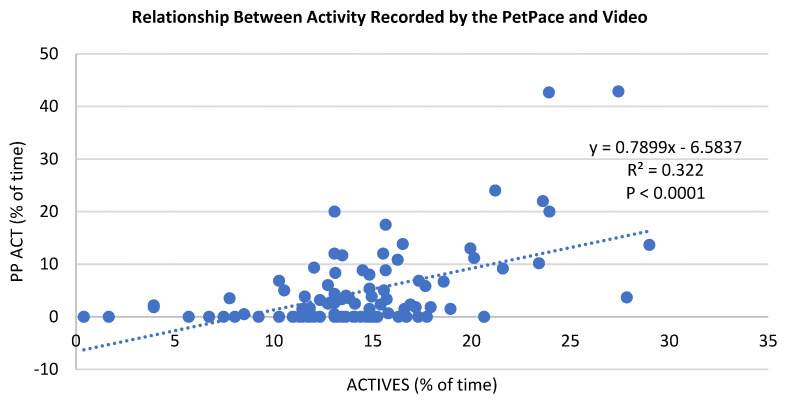
Correlation between activity recorded by the PetPace collar and video (s, % of time). While there is general agreement over a range level of activity, one should not expect PR and activity to be perfectly correlated, as some stressed animals have elevated PR and are inactive, especially when frightened.

By way of validating the PP collars, we calculated correlation coefficients for measures of activity collected by the PP collars compared with video records (Figure 6). While the correlation coefficient (r = 0.56 and R^2^ = 0.322) differed significantly from zero, these two measures of activity are likely measuring different types of activity because of the moderate relationships between the two variables. 

Three cats (37% of cats) from the control group exhibited sickness behaviors (2 vomited and one had excessive salivary secretion) during the transport. No cats in the 2M2B group exhibited sickness behaviors. All cats were adults; cat body weight and age did not interact with treatment effects. 

## 4. Discussion

This study describes a viable model to assess treatment effects in a model for cat transport stress. The study was sensitive enough to detect significant differences within our sample size of eight cats per treatment (*n* = 16). This model is far less variable than when in-home data are collected, especially when conclusions rely on many consumer measures of cat behavior. The PetPace system delivered objective PR data in a reliable fashion. Combining objective behavioral and physiological data gives us the most complete understanding of the effects of a semiochemical.

This is one of the few studies that examined any semiochemical using an objective, randomized, blinded, placebo-controlled model that does not rely on consumer opinion. The placebo effect is very real for animal behavior studies. For example, in Shu and Gu [21]; Figure 3), transported cats given a placebo had a reduced stress score, but the semiochemical treatment group had a still lower stress score (among only cats with high stress scores). This points to the importance of placebo controls, especially in consumer-reported data, in that any effect of an intervention must be greater than any placebo effect. In our work reported here, the effects of 2M2B were larger and more consistent across measures than the data thus far reported for FFS (one of the few semiochemical therapies available). We are not aware of a side-by-side evaluation of these two available semiochemicals in transported cats.

One can examine the measures that indicate that cats with 2M2B collars were less stressed than transported cats with a placebo collar. To the people handling the animals, control-cat stress responses were readily observable. Control cats hid in the back of the kennel, and some control cats vomited or salivated excessively. The objective measures of PR confirmed that cats wearing placebo collars had elevated PR compared with cats with a 2M2B collar. Cats with 2M2B collars also self-groomed (control cats did not) and slept more than cats with placebo-controlled collars. These indicators provide evidence that 2M2B can provide relief to cats during transport or alternative stressful situations. 

Few interventions are available to prevent or reduce stress-induced reactions among domestic cats, apart from drugs. Semiochemicals are not drugs and are natural molecules found in animals and plants. They are considered clean, green, and ethical technologies that are favored over conventional drugs [11]. Semiochemical therapy shows promise for improving the lives of cats in a natural way.

In the current study, cats were exposed to a complex stressor, including confinement in carriers, exposure to an unfamiliar environment (back seat of the vehicle), and the noise, movement, and vibration associated with the movement of the vehicle. While not well studied, a normal reaction of cats to stress is to remain still and inactive; indeed, our control cats spent much time apparently hiding in the back of the transport kennel. Ellis [23] showed that stressed cats prefer concealed areas (hiding), reduce activity levels, and decrease behavior diversity. The following stressors have been reported to cause cats to reduce activity levels: Inconsistent caretakers [24] and novel environments (entering an animal shelter [25]; unfamiliar yards [26]; and visit to veterinary clinic [27]. In this study, 2M2B increased the activity level recorded by the PetPace collar and the video recordings. This finding is interesting because of the limited opportunity for movement in the kennel because cats were confined in the carrier. The findings are also interesting because of the moderate or low correlation between PP and video recording of cat activity. We conclude that live video and the PP collar measure overlapping but not identical measures of cat activity.

According to the videos, cats changed positions (alternating between lying and sitting) and/or repositioned to the back of the carrier when the vehicle started to move or when the road became bumpy (Figure 5). Hiding is a behavior that is exhibited by stressed cats [25,28], and the 2M2B collared cats remained more toward the front of the carrier. With 2M2B collars, transported cats did not hide in the farther back portion of the carrier, where it was darker and more secluded. Cats that wore the 2M2B collars spent more time sleeping and less time sitting than control cats. These behavioral differences exhibited by the control and 2M2B-treated cats during transportation indicated that 2M2B reduced behavioral and physiological signs of stress in cats during transportation.

The 2M2B molecule, although an interomone (not yet able to be called a pheromone), has an effect within seconds to minutes. Note the heart rate data at time −10 and zero time points for cats with control or 2M2B collars. Control cats moved from their kennel to the transport vehicle for a 10 min acclimation period and had about a 12% increase in PR, while cats with 2M2B collars had about a 4% decline in PR during this acclimation period. This means that cats respond to 2M2B as a releaser semiochemical in that its effects are observable in seconds to minutes. However, the effect clearly lasts for much longer (at least the 70 min transport). A collar releasing 2M2B is unlikely to activate the VNO of the cat (unless they scratched the collar and then licked it; a behavior not observed). Thus, the data, while not conclusive, suggest to us that 2M2B activates the MOE rather than the VNO to cause the stress-reducing effects observed.

It is interesting that the effect of 2M2B in reducing stress during transport was apparently greater among male cats than female cats (though females benefited in most measures). The differences between gender results are hard to express currently due to a lack of research. Current studies do not show a difference between behavioral or stress scores between sexes. There is evidence of differences between breeds, but there is not much understanding around gender [29]. Pheromones or interomones that provide alternative results dependent on sex have not been widely studied and offer a new branch of research that should be investigated. The effects of semiochemicals on cats of each sex require further study, and sex-specific therapies may be needed.

## 5. Conclusions

We described here an objective model that examined the stress of transport for individual adult cats. The model is reliable and able to detect biological differences with a sample size of 16 cats (eight per treatment). The model requires multiple vehicles and people to control for effects over time and to avoid contamination of control vehicles with semiochemicals. We show here for the first time that 2M2B can be used to reduce stress-like behavioral and physiological responses among transported cats. 

The overall effects of 2M2B on transported cats revealed a response that was consistent across multiple measures. All measures point to 2M2B providing relief from the behavioral and physiological effects of transport stress. The magnitude of the stress reduction observed here has not been reported for any other intervention to date. We conclude that collars containing 2M2B may serve as a useful tool to reduce stress during transport and perhaps could reduce stress in alternative stressful situations that cats experience. 

## Figures and Tables

**Table 1:** Definitions of electronic measures and ethogram for cat behavioral measures (adapted from [22]).

Measure	Abbreviation	Definition
***Pet Pace data***		
Pulse rate	PR or HR	Cat’s pulse rate of carotid artery, beats per minute (BPM)
Activity score	ACT	Activity density of the cat with scored levels ranging from 0.4 to 25.5, with the arbitrary value being increments of higher activity
***Behavior***		
Lying *	LY	Cat laying with eyes open
Sitting	SIT	Cat adopts sitting position with front limbs supporting the body
Self-grooming	SG	Cat grooming its own body
Sleeping *	SLEEP	Cat laying in a relaxed state with its eyes closed, head down on bedding or on its chest
Adjusting position	ADJUST	Cat adjusting its body because of disturbance by bumpy road and unstable movement of the vehicle or for unknown reasons
Scanned activity	ACTIVITY	The sum of cat grooming itself and adjusting position

* Lying and sleeping were difficult to differentiate at times. Measuring lying behaviors are very accurate; sleeping is more variable in that observer interpretation of eyes being open is required. Of course, cats can close their eyes and not be asleep. Thus, sleeping data are not as reliable as lying behaviors.

**Table 2:** Effects of 2M2B on PetPace measures and behaviors of 16 cats during transport. N = 16 cats.

	Least Squares Means			*p*-Values				
Observations (Definitions in Table 1)	Control	2M2B	SE	TRT	PER	TRT*PER	Sex	TRT*SEX
PR (BPM)	175.82	162.05	1.99	<0.0001	<0.0001	0.64	0.78	0.02
PetPace activity	12.77	14.24	0.57	0.07	<0.0001	0.94	0.004	0.36
LY (% of time)	80.95	74.65	3.80	0.24	0.43	0.01	0.57	0.03
Sleep (% of time)	6.32	18.24	3.02	0.006	0.004	0.36	0.48	0.001
Sit (% of time)	11.54	4.51	2.33	0.04	0.002	0.016	0.0001	0.50
SG (% of time)	0.00	1.77	0.73	0.09	0.31	0.31	0.12	0.12
ACTIVE (% of time) *	1.56	3.02	0.90	0.27	0.77	0.1688	0.60	0.03

* From video records. SE = standard error of the least squares mean. TRT = treatment effect (control vs. 2M2B). PER = period effect; the 8, 10 min periods (see Figure 1). TRT*PER = the interaction between treatment and period. Sex = male vs. female. TRT*Sex = the interaction between treatment and sex.

**Table 3:** Effects of 2M2B on the percentage of time spent on location in the kennel during transport. *n* = 16 cats.

Percentage of Time Spent on Two Locations			
Crate Area	Control	2M2B	Chi-Square
Back	98	53	54.7 *
Front	2	47	

* *p* < 0.001.

## Data Availability

Data are available to qualified scientists upon request.

## Supplementary Materials

The following supporting information can be downloaded at: https://www.mdpi.com/article/10.3390/ani14020341/s1, Table S1: List of cats and their biometric data.

## References

[1] Montague M.J., Li G., Gandolfi B., Khan R., Aken B.L., Searle S.M.J., Minx P., Hillier L.W., Koboldt D.C., Davis B.W. (2014). Comparative Analysis of the Domestic Cat Genome Reveals Genetic Signatures Underlying Feline Biology and Domestication. Proc. Natl. Acad. Sci. USA.

[2] Zhang L., Bian Z., Liu Q., Deng B. (2022). Dealing with Stress in Cats: What Is New about the Olfactory Strategy?. Front. Vet. Sci..

[3] Salazar I., Sánchez-Quinteiro P. (2011). A Detailed Morphological Study of the Vomeronasal Organ and the Accessory Olfactory Bulb of Cats. Microsc. Res. Tech..

[4] Hart B.L., Leedy M.G. (1985). Analysis of the Catnip Reaction: Mediation by Olfactory System, Not Vomeronasal Organ. Behav. Neural Biol..

[5] Reineccius G., Reineccius G. (1994). Flavoring Ingredients Classified As GRAS By the Flavor Extract Manufacturers Association. Source Book of Flavors.

[6] Schaal B., Moncomble A.-S., Langlois D., Schaal B., Rekow D., Keller M., Damon F. (2023). Does the Rabbit Mammary Pheromone Attract Newborns to Maternal Faeces? A New Potential Function of the Suckling Chemosignal. Chemical Signals in Vertebrates 15.

[7] Schaal B., Coureaud G., Langlois D., Giniès C., Sémon E., Perrier G. (2003). Chemical and Behavioural Characterization of the Rabbit Mammary Pheromone. Nature.

[8] Pirner G.M. (2018). Behavioral, Physiological, and Neurological Influences of Pheromones and Interomones in Domestic Dogs. Ph.D. Thesis.

[9] McGlone J.J., Garcia A., Thompson W.G., Pirner G.M. (2019). Maternal-Neonatal Pheromone/Interomone Added to Cat Litter Improves Litter Box Use and Reduces Aggression in Pair-Housed Cats. J. Appl. Anim. Welf. Sci..

[10] McGlone J.J., Thompson G., Devaraj S. (2017). A Natural Interomone 2-Methyl-2-Butenal Stimulates Feed Intake and Weight Gain in Weaned Pigs. Animal.

[11] McGlone J.J., Archer C., Henderson M. (2022). Interpretive Review: Semiochemicals in Domestic Pigs and Dogs. Front. Vet. Sci..

[12] McGlone J.J., Aviles-Rosa E.O., Archer C., Wilson M.M., Jones K.D., Matthews E.M., Gonzalez A.A., Reyes E., Aral F., Payan-Carreira R., Quaresma M. (2021). Understanding sow sexual behavior and the application of the boar pheromone to stimulate sow reproduction. Animal Reproduction in Veterinary Medicine.

[13] McGlone J.J., Thompson W.G., Guay K.A. (2014). CASE STUDY: The Pig Pheromone Androstenone, Acting as an Interomone, Stops Dogs from Barking. Prof. Anim. Sci..

[14] Lewis C.R.G., Hulbert L.E., McGlone J.J. (2008). Novelty Causes Elevated Heart Rate and Immune Changes in Pigs Exposed to Handling, Alleys, and Ramps. Livest. Sci..

[15] Mariti C., Guerrini F., Vallini V., Bowen J.E., Fatjó J., Diverio S., Sighieri C., Gazzano A. (2017). The Perception of Cat Stress by Italian Owners. J. Vet. Behav..

[16] Tateo A., Nanni Costa L., Padalino B. (2022). The Welfare of Dogs and Cats during Transport in Europe: A Literature Review. Ital. J. Anim. Sci..

[17] Kronen P.W., Ludders J.W., Erb H.N., Moon P.F., Gleed R.D., Koski S. (2006). A Synthetic Fraction of Feline Facial Pheromones Calms but Does Not Reduce Struggling in Cats before Venous Catheterization1. Vet. Anaesth. Analg..

[18] Mills D.S., Redgate S.E., Landsberg G.M. A Meta-Analysis of Studies of Treatments for Feline Urine Spraying, 2011. https://journals.plos.org/plosone/article?id=10.1371/journal.pone.0018448.

[19] Cozzi A., Lecuelle C.L., Monneret P., Articlaux F., Bougrat L., Mengoli M., Pageat P. Induction of Scratching Behaviour in Cats: Efficacy of Synthetic Feline Interdigital Semiochemical—Alessandro Cozzi, Céline Lafont Lecuelle, Philippe Monneret, Florence Articlaux, Laurent Bougrat, Manuel Mengoli, Patrick Pageat, 2013. https://journals.sagepub.com/doi/full/10.1177/1098612X13479114.

[20] Wyatt T.D. (2017). Pheromones. Curr. Biol..

[21] Effect of a Synthetic Feline Facial Pheromone Product on Stress during Transport in Domestic Cats: A Randomised Controlled Pilot Study—Hang Shu, Xianhong Gu, 2022. https://journals.sagepub.com/doi/full/10.1177/1098612X211041305.

[22] May M.D., Surowiec K., McGlone J.J. (2015). Vapor Release of 2-Methyl-2-Butenal from Dog Collars and Spray Containing Pheromone or Interomone Measured by Solid-Phase Microextraction and Gas Chromatography-Mass Spectrometry. Prof. Anim. Sci..

[23] Ellis S.L. (2009). Environmental Enrichment: Practical Strategies for Improving Feline Welfare. J. Feline Med. Surg..

[24] Carlstead K., Brown J.L., Strawn W. (1993). Behavioral and Physiological Correlates of Stress in Laboratory Cats. Appl. Anim. Behav. Sci..

[25] Kry K., Casey R. (2007). The Effect of Hiding Enrichment on Stress Levels and Behaviour of Domestic Cats (*Felis sylvestris catus*) in a Shelter Setting and the Implications for Adoption Potential. Anim. Welf..

[26] Rehnberg L.K., Robert K.A., Watson S.J., Peters R.A. (2015). The Effects of Social Interaction and Environmental Enrichment on the Space Use, Behaviour and Stress of Owned Housecats Facing a Novel Environment. Appl. Anim. Behav. Sci..

[27] Nibblett B.M., Ketzis J.K., Grigg E.K. (2015). Comparison of Stress Exhibited by Cats Examined in a Clinic versus a Home Setting. Appl. Anim. Behav. Sci..

[28] Vinke C.M., Godijn L.M., van der Leij W.J.R. (2014). Will a Hiding Box Provide Stress Reduction for Shelter Cats?. Appl. Anim. Behav. Sci..

[29] Fazio E., Ferlazzo A., Cravana C., Medica P. (2017). Comparison of Acute versus Chronic Stress Responses to Different Housing’s Systems of Cats. Acta Sci. Vet..

