# Supplementary material for: Semiochemical 2-Methyl-2-butenal Reduced Signs of Stress in Cats during Transport
Source: Animals (Basel). 2024 Jan 22;14(2):341. doi: 10.3390/ani14020341 (PMC10812570; doi:10.3390/ani14020341)
Supplement: Supplementary file 1 [file animals-14-00341-s001.zip › animals-2769418-supplementary.pdf]

Supplementary 1 (S1)

Table S1. List of cats and their sex, weight and age used in this study

Cat ID	Sex	Body weight, kg	Age (year)	Trip	TRT
241F	F	2.6	2	1	Control
245M	M	3.7	1.5	2	Control
217M	M (castrated)	4.4	9	3	Control
215F	F	2.8	9	4	Control
23M	M	4.7	16	1	Control
52F	F	3.8	10	2	Control
251M	M	4.4	1.5	3	Control
13CPU5F	F	2.9	2	4	Control
249F	F (spayed)	3.3	9	1	2M2B
242M	M	4.6	2	2	2M2B
234F	F	4.7	6	3	2M2B
221M	M	3.4	6	4	2M2B
239M	M	4.7	2	1	2M2B
250F	F	3.8	1.5	2	2M2B
248M	M	4.3	1.5	3	2M2B
246F	F	4.5	1.5	4	2M2B
